# Identification of a gonadotropin-releasing hormone receptor orthologue in *Caenorhabditis elegans*

**DOI:** 10.1186/1471-2148-6-103

**Published:** 2006-11-29

**Authors:** Sivan Vadakkadath Meethal, Miguel J Gallego, Ryan J Haasl, Stephen J Petras, Jean-Yves Sgro, Craig S Atwood

**Affiliations:** 1 Section of Geriatrics and Gerontology, Department of Medicine, University of Wisconsin and Geriatric Research, Education and Clinical Center, Veterans Administration Hospital, Madison, WI 53705, USA; 2 Biotechnology Center, University of Wisconsin, Madison, WI 53705, USA; 3 Case Western Reserve University, Cleveland, OH 44106, USA

## Abstract

**Background:**

The *Caenorhabditis elegans *genome is known to code for at least 1149 G protein-coupled receptors (GPCRs), but the GPCR(s) critical to the regulation of reproduction in this nematode are not yet known. This study examined whether GPCRs orthologous to human gonadotropin-releasing hormone receptor (GnRHR) exist in *C. elegans*.

**Results:**

Our sequence analyses indicated the presence of two proteins in *C. elegans*, one of 401 amino acids [GenBank: NP_491453; WormBase: F54D7.3] and another of 379 amino acids [GenBank: NP_506566; WormBase: C15H11.2] with 46.9% and 44.7% nucleotide similarity to human GnRHR1 and GnRHR2, respectively. Like human GnRHR1, structural analysis of the *C. elegans *GnRHR1 orthologue (Ce-GnRHR) predicted a rhodopsin family member with 7 transmembrane domains, G protein coupling sites and phosphorylation sites for protein kinase C. Of the functionally important amino acids in human GnRHR1, 56% were conserved in the *C. elegans *orthologue. Ce-GnRHR was actively transcribed in adult worms and immunoanalyses using antibodies generated against both human and *C. elegans *GnRHR indicated the presence of a 46-kDa protein, the calculated molecular mass of the immature Ce-GnRHR. Ce-GnRHR staining was specifically localized to the germline, intestine and pharynx. In the germline and intestine, Ce-GnRHR was localized specifically to nuclei as revealed by colocalization with a DNA nuclear stain. However in the pharynx, Ce-GnRHR was localized to the myofilament lattice of the pharyngeal musculature, suggesting a functional role for Ce-GnRHR signaling in the coupling of food intake with reproduction. Phylogenetic analyses support an early evolutionary origin of GnRH-like receptors, as evidenced by the hypothesized grouping of Ce-GnRHR, vertebrate GnRHRs, a molluscan GnRHR, and the adipokinetic hormone receptors (AKHRs) and corazonin receptors of arthropods.

**Conclusion:**

This is the first report of a GnRHR orthologue in *C. elegans*, which shares significant similarity with insect AKHRs. In vertebrates, GnRHRs are central components of the reproductive endocrine system, and the identification of a GnRHR orthologue in *C. elegans *suggests the potential use of *C. elegans *as a model system to study reproductive endocrinology.

## Background

G protein-coupled receptors (GPCRs) are ancient molecules that act as vital sensors of environmental and internal physiological signals in organisms. This family of proteins which forms the largest class of cell surface receptors found in animal genomes [1,2], has an early evolutionary origin [3-6], and serves a wide variety of functions including reproduction. Structurally, all known GPCRs share a common architecture of seven membrane-spanning helices connected by intra- and extracellular loops.

*C. elegans *is a simple, highly reproductive, multicellular model organism appropriate to the investigation of innumerable signaling pathways at the organismal level. Despite our knowledge of the reproductive physiology of *C. elegans*, the molecular endocrinology regulating reproduction in *C. elegans *is unknown. The *C. elegans *genome is known to code for at least 1149 GPCRs [6] but the GPCR(s) critical to the regulation of reproduction in this nematode are not yet known. The characterization of membrane receptors related to the regulation of reproduction within this model nematode organism is very important for both the study of evolutionary biology as well as the study of the molecular endocrinology of reproduction in multicellular organisms.

In mammals, reproduction is controlled by hormones of the hypothalamic-pituitary-gonadal (HPG) axis and hostile environmental conditions are known to suppress HPG axis hormones, thereby decreasing or preventing reproduction [7]. The hypothalamus acts as a sensor of the environment to regulate the production of gonadotropin-releasing hormone (GnRH1). GnRH1 released from hypothalamic neurons into the hypophyseal bloodstream binds to GnRH receptors (GnRHR1) on gonadotropes of the anterior pituitary signaling for the synthesis and secretion of gonadotropins. Gonadotropins in turn bind to receptors on the gonads leading to the production of the sex steroids [8]. The presence of a complex endocrine axis that regulates reproduction in *C. elegans *has not been contemplated, since central components of this axis – gonadotropin-releasing hormone receptor (GnRHR) and its ligand(s) – have not been reported.

In this study we demonstrate that *C. elegans *contains a GnRHR (Ce-GnRHR) orthologous to GnRHR1 in humans and to the adipokinetic hormone receptors (AKHRs) of insects, and that Ce-GnRHR specifically localizes to the nuclei of germline and intestinal cells, and to the myofilament lattice of the pharyngeal musculature. Our results support the presence of an evolutionarily conserved GPCR possibly involved in reproduction and metabolism in *C. elegans*.

## Results

### Sequence analysis

**Figure 1:**
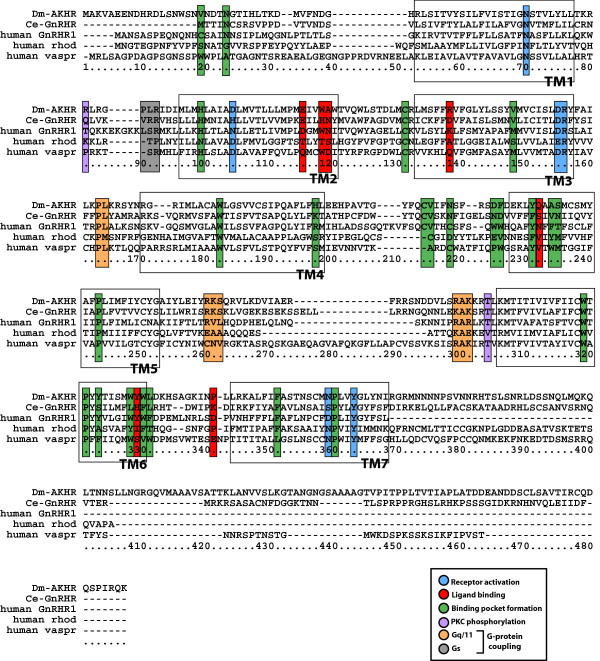
**Identification of GnRHR orthologue in *C. elegans***. Protein sequence alignment of *Drosophila melanogaster *AKHR [GenBank: AAC61523], Ce-GnRHR [GenBank: NP_491453], human GnRHR1 [GenBank: NP_000397], human rhodopsin [GenBank: NP_000530], and human vasopressin type 1A receptor [GenBank: NP_000697]. Alignment was generated in ClustalX using default gap penalties. Colored boxes indicate functionally important residues (FIRs) in human GnRHR1 and their putative homologues in the other four sequences. Similarity analysis of these FIRs is presented in Table 2. Open boxes delimit the seven transmembrane domains of human GnRHR1.

**Figure 2:**
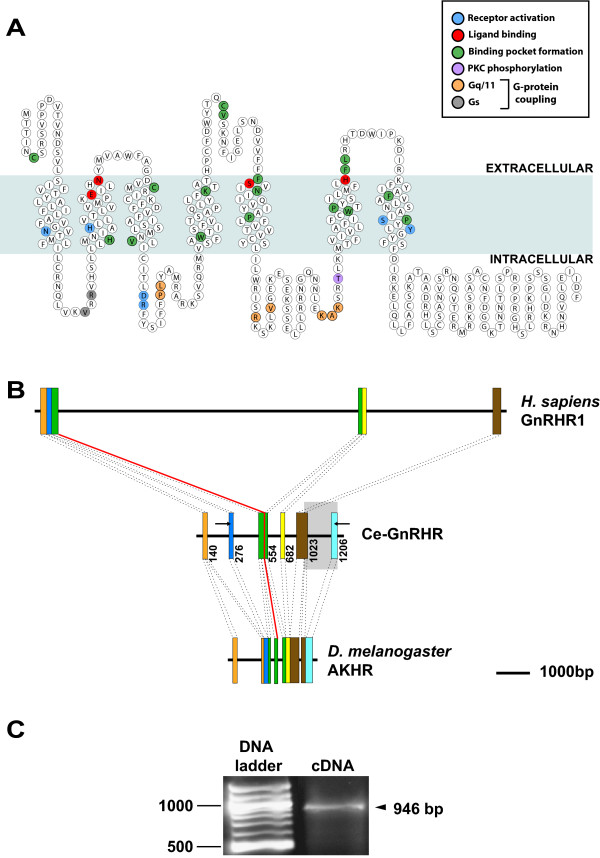
**Structural organization of Ce-GnRHR and detection of Ce-GnRHR mRNA**. (A). Two-dimensional representation of the Ce-GnRHR showing the conserved, functionally important, amino acid residues. Predictions were made according to SOSUI [65]. Putative ligand binding sites, and residues important in receptor activation, binding pocket formation, G-protein coupling and PKC phosphorylation are indicated in the figure legend. These functionally important residues were derived from the predicted structure of human GnRHR1 by Miller et al. [11]. (B). Comparative genomic organization of human GnRHR1, Ce-GnRHR, and D. melanogaster AKHR (Dm-AKHR). Exons are represented by tall, colored boxes. Exon colors in human GnRHR1 and Dm-AKHR sequences correspond to homologous regions in the Ce-GnRHR sequence. The gray box delimits the C-terminus portion of Ce-GnRHR absent from human GnRHR1, while the red line traces the correspondence between the ends of human GnRHR1 exon 1 and Dm-AKHR exon 3. Arrows superimposed upon Ce-GnRHR represent the locations of the forward and reverse primers used to amplify Ce-GnRHR cDNA. Numbers to the right of Ce-GnRHR exons indicate the mRNA nucleotide upon which the preceding exon terminates. (C). Ce-GnRHR mRNA expression. Gel showing the 946 bp Ce-GnRHR cDNA derived from RT-PCR using the forward and reverse primers shown in (B) above (see details of RNA isolation and RT-PCR in Methods). The DNA ladder is indicated in bp on the left. Alignment of the fragment sequence with *C. elegans *genomic sequence confirmed the synthesized fragment originated from the Ce-GnRHR mRNA template.

Sequence similarity searches using the sequences of the principal GPCR signaling components of the human HPG axis were performed against the *C. elegans *genome. This analysis indicated the presence of two proteins, one of 401 amino acids [GenBank: NP_491453] and another of 379 amino acids [GenBank: NP_506566] sharing 46.9% and 44.7% nucleotide similarity to that of human GnRHR1 and GnRHR2, respectively (Table 1). In addition, a leucine-rich GPCR previously reported in *C. elegans *[GenBank: NM_073147] has 47.6% nucleotide similarity to human follicle stimulating hormone receptor (FSHR) [9]. This paper is primarily focused on the 401 amino acid protein [GenBank: NP_491453] orthologous to human GnRHR1. Amino acid sequence alignment of human GnRHR1 with the *C. elegans *orthologue (Ce-GnRHR) and 3 other class A GPCRs demonstrated considerable sequence similarity (Fig. 1). Like its human orthologue, 3 separate prediction algorithms used to analyze the structure of Ce-GnRHR predicted a GPCR belonging to the rhodopsin family (Figs. 1 and 2A; See additional file 1: TM_predictions.eps; [10]). These prediction programs indicate that Ce-GnRHR contains several structural motifs similar to that of human GnRHR1, including 7 transmembrane domains, 3 intracellular and extracellular loops, and amino acid residues representing PKC phosphorylation sites and G-protein coupling sites (Figs. 1 and 2A; See additional file 1: TM_predicitions.eps; [11]). As has been reported for all non-human GnRHR1s [11], the Ce-GnRHR has a long C-terminal tail (Figs. 1 and 2A) characteristic of a gene ancestral to GnRH and AKH receptors. Although the overall amino acid sequence identity between *C. elegans *and human GnRHR1 was only ~21%, closer analysis revealed that 56% of the functionally important amino acid residues (FIRs) of human GnRHR1 were conserved in Ce-GnRHR (Figs. 1 and 2A; Table 2). The highest degree of conservation was observed in those amino acids involved in receptor activation (83%), and G_q11 _coupling (62%; Table 2). Lesser conservation was observed in amino acids important to binding pocket formation (54%), G_s _coupling (50%), and PKC phosphorylation (50%). Ligand binding sites were least conserved (36%), indicating the possibility that the ligand of Ce-GnRHR is significantly different from that of human GnRHR1. Indeed, in *Drosophila melanogaster*, a cloned GnRHR [12] was subsequently identified as AKHR from ligand binding studies indicating AKH was the binding partner of this receptor in insects [13]. In this connection, Ce-GnRHR had the highest overall sequence similarity (45%) and the highest FIR similarity (66%) with *Drosophila melanogaster *AKHR (Dm-AKHR; Table 2; [13]), a receptor involved in metabolism (Table 2). In comparison, there was less conservation of overall sequence and FIRs in two other human class A GPCRs, rhodopsin (12% and 41%; respectively) and vasopressin receptor type 1A (18% and 44%; respectively) when compared with *C. elegans *(21% and 56%; respectively; Table 2). This comparative analysis suggests Ce-GnRHR is orthologous to insect AKHR and human GnRHR. The relatively greater conservation of FIRs observed between Ce-GnRHR, Dm-AKHR and human GnRHR1 (56–66% similarity; Table 2), suggests these proteins are *all *derived from a single ancestral GPCR, and, are thus orthologous to each other.

**Table 1:** *C. elegans *orthologues of GnRH and FSH receptors.

**HPG hormone receptor**	**Gene**	**Length**		**% Identity**		**Mass (kDa)**
		*Nucleotide*	*Amino acid*	*Nucleotide*	*Amino acid*	
GnRH Receptor 1	AF039712	1206	401	46.9	22.2	46.2
GnRH Receptor 2	NM_074165	1140	379	44.7	18.5	43.5
*FSH Receptor	NM_073147	2790	929	47.6	25.4	104

The percent nucleotide and amino acid identity of *C. elegans *orthologues was determined using ALIGN. % identity is derived from the ALIGN pairwise alignment. Mass is calculated from the amino acid sequence. *Identification of the FSH receptor orthologue as reported in [9]; percent identities for this receptor were derived from the total alignment of the orthologue and human FSHR.

**Table 2:** Conservation of functionally important amino acid residues (FIRs).

**Functional Site Type **(# of residues)	Ce-GnRHR vs. human GnRHR1	Ce-GnRHR vs. Dm-AKHR	Human GnRHR1 vs. Dm-AKHR	Ce-GnRHR vs. human Rhodopsin	Ce-GnRHR vs. human Vasopressin receptor
Receptor activation (6)	83.3%	83.3%	83.3%	75.0%	83.3%
Ligand binding (7)	35.7%	21.4%	42.9%	7.1%	35.7%
Binding pocket formation (24)	54.2%	68.8%	72.9%	43.8%	33.3%
PKC phosphorylation (2)	50.0%	75.0%	50.0%	25.0%	50.0%
G_q_/11 G-protein coupling (8)	62.5%	93.8%	68.8%	56.2%	62.5%
G_s _G-protein coupling (3)	50.0%	33.3%	33.3%	0.0%	33.3%
**Total similarity (FIRs only)**	**56.0%**	**66.0%**	**66.0%**	**41.0%**	**44.0%**

**Identity (all residues)**	**20.8%**	**26.6%**	**20.4%**	**12.4%**	**17.9%**
**Identity + Similarity (all residues)**	**36.3%**	**45.2%**	**37.9%**	**28.5%**	**34.4%**

Shown are the amino acid similarities between the functionally important residues of Ce-GnRHR and human GnRHR1, *Drosophila melanogaster *AKHR (Dm-AKHR), human rhodopsin, and human vasopressin type 1a. Also shown are the overall amino acid identity/similarity for each comparison. 'Similarity' of compared amino acids was based on the BLOSUM62 matrix, a more conservative measure of similarity than that used in the ALIGN algorithm, and percentages were calculated as described in methods.

Despite the orthology of these sequences, the genomic organization of Ce-GnRHR, human GnRHR1, and Dm-AKHR are radically different (Fig. 2B). The coding region of each gene contains a different number of exons: 6 in Ce-GnRHR, 5 in Dm-AKHR, and 3 in human GnRHR1. Intronic sequence length is also variable. While the 2 introns of human GnRHR1 total more than 10 kilobases, the 4 introns of Dm-AKHR only amount to ~1 kilobase. Additionally, sequence corresponding to the 3' end of Ce-GnRHR exon 5 and all of Ce-GnRHR exon 6 is absent from human GnRHR. In order to deduce the genomic organization of a gene ancestral to all three of these GnRHR(-like) genes, a considerably larger number of genes would have to be analyzed. Yet, at least one coincidental feature of the gene maps shown in Fig. 2B might be indicative of ancestral gene structure: exon 1 of human GnRHR and exon 3 of DM-AKHR terminate at the same point.

### Identification and immunolocalization of Ce-GnRHR

To determine whether Ce-GnRHR was transcribed in the worm, we isolated RNA, and, using two gene-specific primers, amplified the region depicted in Fig. 2B. The expected 946 bp cDNA fragment encompassing exons 2 through 6 was detected (Fig. 2C). The sequence of the amplified cDNA [GenBank; NM_059052], matched the genomic sequence (chromosome 1; [GenBank: NC_003279]), minus intronic sequence, demonstrating that the amplified cDNA was amplified from Ce-GnRHR mRNA template. This confirms that Ce-GnRHR is actively transcribed in adult *C. elegans*.

**Figure 3:**
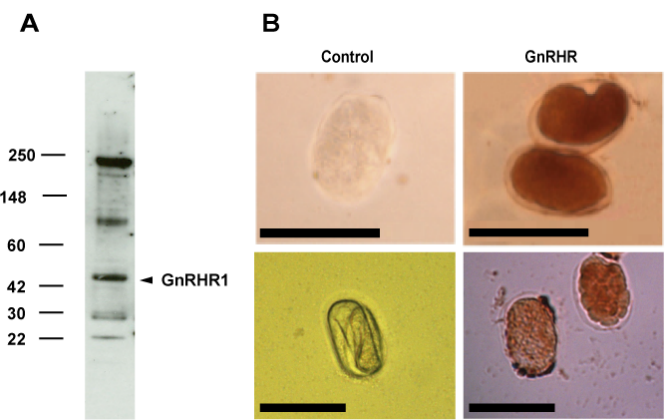
**Expression of Ce-GnRHR**. (A). Worm (N2) homogenates were immunoblotted with an affinity purified polyclonal antibody against Ce-GnRHR. The antibody recognized a 46-kDa band, the calculated molecular weight of the GnRHR1 orthologue. The secondary antibody alone did not show immunoreactivity. The molecular weight markers are indicated in kDa on the left. (B). Immunohistochemical localization of Ce-GnRHR was performed on eggs after freeze fracture using a monoclonal antibody against human GnRHR1 (above) or an affinity purified polyclonal antibody against Ce-GnRHR (below). Eggs were similarly stained by both antibodies. No immunoreactivity was detected in eggs probed with the secondary antibody alone. Scale: eggs – 50 μm.

To determine whether Ce-GnRHR protein was expressed in *C. elegans*, we generated a polyclonal antibody against the C-terminus (amino acids 386 to 401) of Ce-GnRHR and the nematode homogenate was subjected to immunoblot analyses. The affinity purified anti-Ce-GnRHR antibody recognized a 46-kDa band (Fig. 3A), the calculated molecular weight of Ce-GnRHR, as well as higher molecular weight (116-kDa, 237-kDa) variants that might represent mature or otherwise post-translationally modified species and lower molecular weight (27-kDa and 23-kDa) variants that might represent truncated protein products, as we have reported for human GnRHR1 [14]. Despite limited similarity between the N-terminus of the human and *C. elegans *receptor, a monoclonal antibody raised against the N-terminus of human GnRHR1 identifies a single band of 46-kDa in the *C. elegans *homogenate (See [Supplementary-material S2]: Immunoblot 1.eps) and produces similar immunolocalization (see below); however, it remains unclear whether this antibody is detecting Ce-GnRHR. The specificity of the polyclonal antibody against Ce-GnRHR for Ce-GnRHR compared to the putative orthologue to human GnRHR2 [GenBank; NP_506566] (orthologue 2) is indicated by the fact that the antibody does not recognize a band at 43.5-kDa, the molecular weight of the immature form of orthologue 2 (Fig. 3A). Additionally, comparison of the amino acid sequences and genomic organization of Ce-GnRHR and orthologue 2 revealed limited similarity between the two genes, especially at the C-terminus of Ce-GnRHR – against which the anti-Ce-GnRHR antibody was raised (See additional file 3: Ortho.eps).

**Figure 4:**
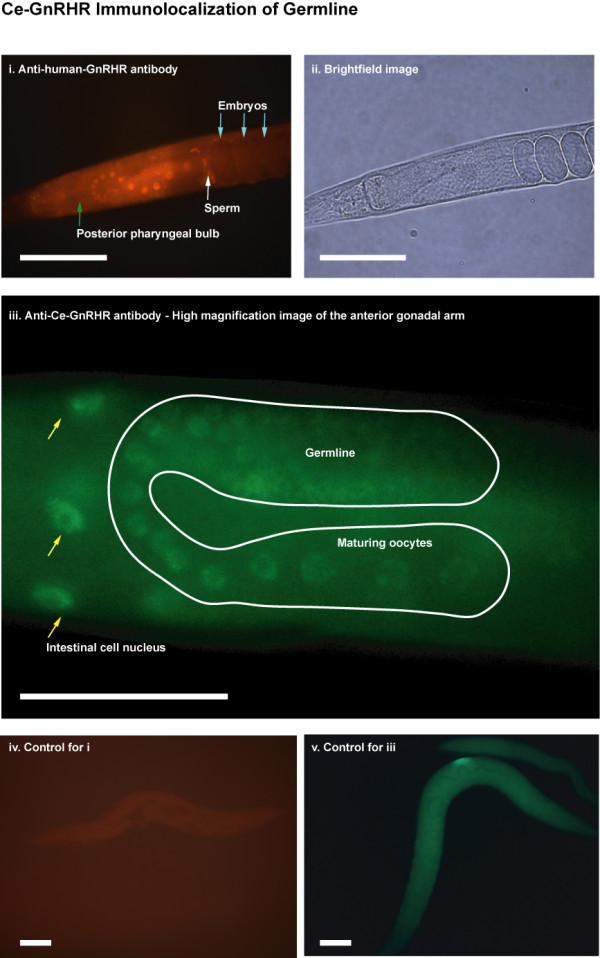
**Immunofluorescent localization of Ce-GnRHR to the germline**. (i). Permeabilized adult N2 worm probed with an anti-human GnRHR1 monoclonal antibody. Anterior view illustrating apparent nuclear immunostaining of maturing oocytes, and sperm. (ii). Brightfield image of same worm shown in (i). (iii). Immunofluorescent localization of GnRHR in a permeabilized adult worm using a polyclonal antibody raised against Ce-GnRHR. High magnification image of anterior gonadal arm (highlighted in white) illustrates nuclear staining of maturing oocytes, and intestinal (yellow arrows) cells. Immunofluorescent staining clearly illustrates an increase in the size of the oocyte nuclei as they mature along the gonadal arm. (iv). Permeabilized adult worm probed with rhodamine-coupled secondary antibody (control for anti-human GnRHR1 antibody staining). (v). Permeabilized adult worm probed with FITC-coupled secondary antibody (control for anti-Ce-GnRHR antibody staining). No specific immunoreactivity was detected with either secondary antibody alone. Scale (i, ii): 100 μm., Scale (iii-v): 50μm.

To examine the localization of Ce-GnRHR, eggs were immunostained with the monoclonal antibody against human GnRHR1, or the anti-Ce-GnRHR antibody (Fig. 3B). Both the N-terminus (anti-human-GnRHR1 antibody) and C-terminus (anti-Ce-GnRHR antibody) antibodies immunolabeled the same structures (see also later, Fig. 4), indicating that Ce-GnRHR is expressed in eggs. With both antibodies, intense staining was observed within the egg, but not the cuticle (Fig. 3B). Staining was not apparent in eggs treated with secondary antibody alone. The authenticity of the anti-human GnRHR1 antibody was verified by positive staining of gonadotropes in sections of human pituitary (data not shown).

To better localize cellular staining in worms, we permeabilized worms and performed whole-mount fluorescent immunohistochemistry. These experiments indicated that Ce-GnRHR was localized to the nucleus of maturing oocytes and intestinal cells (Fig. 4i–iii), to sperm, pharyngeal muscles (see later), but not other cells such as hypodermal cells. Similar staining of these structures was evident for both the anti-human-GnRHR1 and anti-Ce-GnRHR antibodies. Ce-GnRHR staining clearly illustrates an increase in the size of the oocyte nuclei as they mature along the gonadal arm (Fig. 4i, 4iii). Upon fertilization, Ce-GnRHR staining becomes diffuse throughout the developing egg, although staining appears to increase during egg development (see Figs. 3B for staining of laid eggs). Only uniform background autofluorescence was apparent in worms treated with secondary antibody alone (Figs. 4iv, 4v).

**Figure 5:**
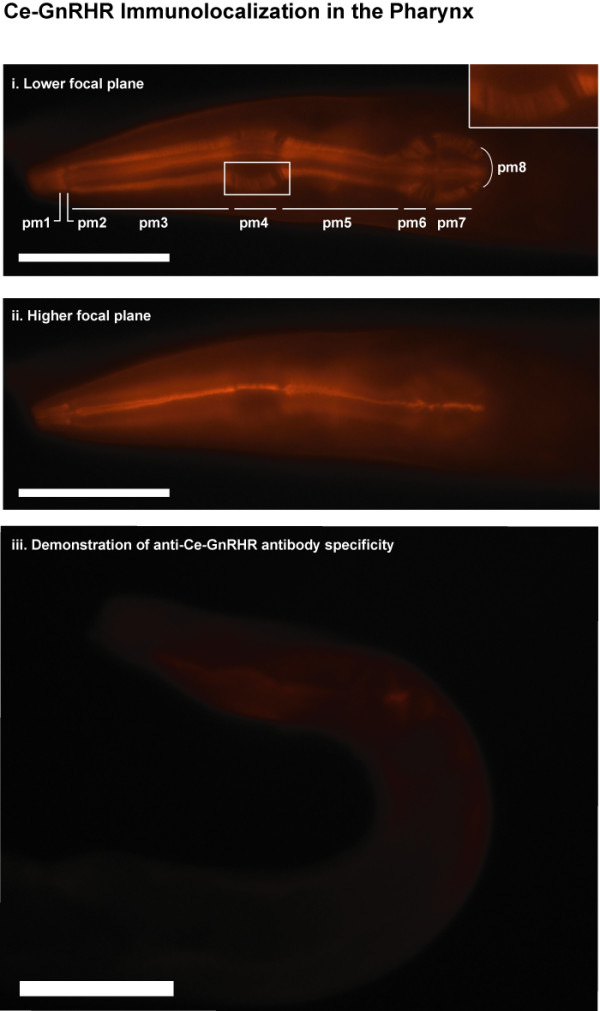
**Immunolocalization of Ce-GnRHR to the pharynx**. (i). Permeabilized adult N2 worm probed with anti-Ce-GnRHR antibody. The myofilament lattice of the pharyngeal muscles were stained intensely with anti-Ce-GnRHR antibody. Immunostaining was localized along the three parallel muscles that comprise the pharyngeal musculature beginning at the tip of the head and extending to the pharyngeal bulb. All 8 pharyngeal muscle domains (pm 1–8) are stained (the focal plane is nearly at the center of the body axis such that all three domains are seen separately). The gap between contractile zones in two adjacent pharyngeal muscle territories are evident. Myofilaments running radially are most obvious in the bulb (see enlarged view in insert). (ii). Higher focal plain of worm in (i) showing more pronounced lattice of one myofilament. (iii). Pre-incubation of anti-Ce-GnRHR antibody with its antigen peptide abolished staining in permeabilized adult worms, demonstrating the specificity of the antibody for Ce-GnRHR. Scale: 50μm.

Interestingly, Ce-GnRHR also was detected along the myofilament lattice of the pharyngeal muscles as evidenced by 1). staining along the three parallel muscles that comprise the pharyngeal musculature beginning at the tip of the head and extending to the pharyngeal bulb (Fig. 5i; taken near the center of the body axis), 2). staining of all 8 pharyngeal muscle (pm1-8) domains, but not in the gaps between contractile zones of adjacent pharyngeal muscle domains (Fig. 5i), and 3). staining of myofilaments that run radially towards the lumen and which are most obvious in the bulbs (Fig. 5i; see online Handbook of Worm Anatomy [15] and [16] for details of pharyngeal anatomy). In Fig. 5ii taken at a higher focal plain, only one muscle lattice is observed (radial filaments are seen "end-on" pointing downward into the plane of section towards the lumen). These results indicate Ce-GnRHR is present on the pharyngeal musculature.

The specificity of binding of anti-Ce-GnRHR antibody to GnRHR was demonstrated by the lack of staining throughout the worm (including the germline, pharyngeal muscle and intestinal cells) when the antibody was preincubated with its antigen (the C-terminus amino acids 386 to 401 as described in the Methods; Fig. 5iii).

**Figure 6:**
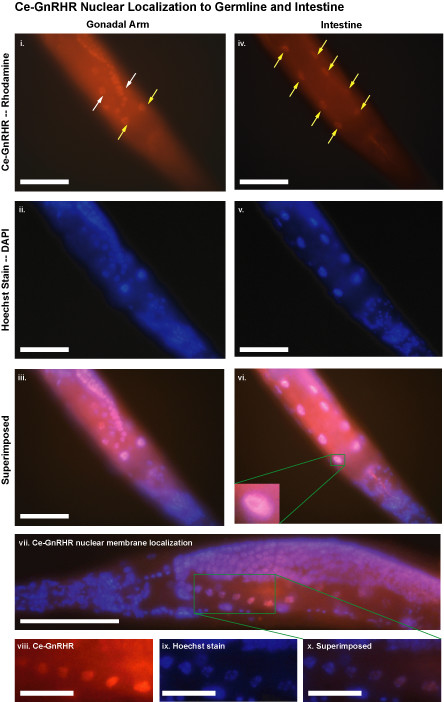
**Nuclear localization of Ce-GnRHR to the germline and intestinal cells**. Permeabilized adult N2 worm probed with anti-Ce-GnRHR antibody (i) and Hoechst stain (ii). Nuclear localization of Ce-GnRHR to oocytes was clearly demonstrated following superimposition of images i and ii (iii). Permeabilized adult N2 worm probed with anti-Ce-GnRHR antibody (iv) and Hoescht stain (v). Nuclear localization of Ce-GnRHR to the intestinal cells was clearly demonstrated following superimposition of images iv and v (vi). A high magnification image of a superimposed nucleus is presented in the insert of vi. A superimposed image of the gonadal arm of an adult N2 worm is shown in vii. Higher magnification images of the gonadal arm stained with anti-Ce-GnRHR antibody (viii), Hoechst stain (ix) and superimposed (x) demonstrates Ce-GnRHR staining is primarily confined to the nuclear membrane. Scale: 50μm.

To confirm the nuclear localization of Ce-GnRHR, we stained *C. elegans *with Ce-GnRHR antibody and a nuclear stain (Hoechst dye) and superimposed the images. There was a clear overlap between Ce-GnRHR immunoreactivity and the nuclear stain in both oocytes and intestinal cell nuclei (Fig. 6i–vi), demonstrating that Ce-GnRHR was localized to the nuclear membrane of oocytes and intestinal cells. At higher magnification, separate chromosomes are evident with the Hoechst stain (Fig. 6ix) while Ce-GnRHR staining is uniform (Fig. 6viii), suggesting that Ce-GnRHR stains the membrane rather than intranuclear structures. This was again demonstrated by the scattered color of the superimposed image (Fig. 6x). In summary, these results indicate that Ce-GnRHR is present on the nuclear membrane of oocytes and intestinal cells. Further, these results corroborate earlier reports of GnRHR1 localization to the nucleus of proliferating cells [17,18].

### Phylogenetic analysis

**Figure 7:**
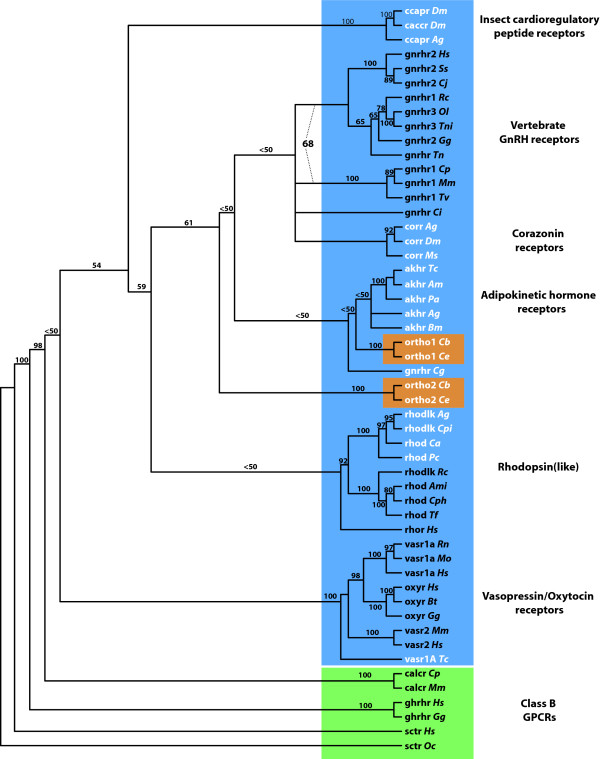
**Unrooted strict consensus tree of 3 equally parsimonious gene trees (1000 random sequence additions with TBR)**. Bootstrap proportions (500 bootstrap replicates, 10 random sequence additions each) for each hypothesized receptor group are indicated above or below the branch leading to the group. Bootstrap proportions for hypothesized receptor groups are provided only if greater than 50%. The 68% bootstrap proportion indicated by dashed lines corresponds to the entire group of vertebrate GnRHRs, a group not represented in the strict consensus tree. Class A GCPRs are shaded in blue, class B GPCRs are shaded in green, nematode orthologues are shaded in orange, vertebrate receptor sequence labels are black and invertebrate receptor sequence labels are white. Tree is derived from a 422 amino acid alignment of Class A and B GPCRs (See additional file 4: GPCRs_alignment.pdf). **GPCR abbreviations**: akhr *Adipokinetic hormone receptor*; caccr *Cardioacceleratory peptide receptor*; calcr *Calcitonin receptor*; ccapr *Crustacean cardioactive peptide receptor*; corr *Corazonin receptor*; ghrhr *Growth hormone releasing hormone receptor*; gnrhr *Gonadotropin releasing hormone receptor (types 1, 2, and 3)*; ortho1 *AKHR/GnRHR orthologue 1*; *ortho2 AKHR/GnRHR orthologue 2*; oxyr *Oxytocin receptor*; rhodlk *Rhodopsin-like*; rhod *Rhodopsin*; sctr *Secretin receptor*; vasr *Vasopressin receptor (types 1A and 2)*. **Organism abbreviations**: Af *Astyanax fasciatus *(teleost fish); Ag *Anopheles gambiae *(mosquito); Am *Apis mellifera *(bee); Ami *Alligator mississippiensis *(alligator); Bm *Bombyx mori *(moth); Bt *Bos Taurus *(cattle); Ca *Camponotus abdominalis *(ant); Cb *Caenorhabditis briggsae *(nematode); Ce *Caenorhabditis elegans *(nematode); Cg *Crassostrea gigas *(oyster); Ci *Ciona intestinalis *(tunicate); Cj *Callithrix jacchus *(marmoset); Cp *Cavia porcellus *(guinea pig); Cph *Caluromys philander *(opossum); Cpi *Culex pipiens *(mosquito); Dm *Drosophila melanogaster *(fruit fly); Gg *Gallus gallus *(chicken); Hs *Homo sapiens *(human); Mm *Mus musculus *(mouse); Mo *Microtus ochrogaster *(vole); Ms *Manduca sexta *(moth); Oc *Oryctolagus cuniculus *(rabbit); Ol *Oryzias latipes *(teleost fish); Pa *Periplaneta americana *(cockroach); Pc *Procambarus clarkii *(crustacean); Rc *Rana catesbeiana *(bull frog); Rn *Rattus norvegicus *(rat); Ss *Sus scrofa *(pig); Tc *Tribolium castaneum *(beetle); Tn *Typhlonectes natans *("rubber eel", amphibian); Tni *Tetraodon nigroviridis *(teleost fish); Tv *Trichosurus vulpecula *(opossum).

Maximum parsimony analysis of the Class A and B GPCR dataset (422 amino acids; See additional file 4: GPCRs_alignment.pdf) yielded 3 most parsimonious trees, the strict consensus of which resulted in the collapse of only 3 nodes (Fig. 7). Relatively robust support (68% boostrap proportion) was found for a group comprising all 3 vertebrate GnRHR types, and, though not achieving strong bootstrap support, all most parsimonious trees contained a group comprising tunicate GnRHR (*Ciona intestinalis*), insect corazonin receptors, and all vertebrate GnRHRs. Recovered in all of the most parsimonious trees, a monophyletic group of AKHRs was hypothesized as the sister group to the group Ce-GnRHR + *C. briggsae*-GnRHR. Together with the molluscan GnRHR (*Crassotrea gigas*), these receptors were hypothesized to be the sister group to the group of corazonin receptors + chordate GnRHRs discussed above. The group of all GnRHRs and GnRHR-like receptors was recovered more often than not (61% bootstrap proportion), to the exclusion of other Class A GPCRs included in the analysis.

## Discussion

Our results demonstrate for the first time the presence of a GPCR in the nematode *C. elegans *with homology to human GnRHR1 and AKHRs of insects (Figs. 1, 2, 3, 4, 5, 6; Tables 1 &2). The nematode GPCR superfamily consists of 170 rhodopsin-like receptors, 650 seven-TM chemoreceptors, and other similar proteins, and represents the largest gene family accounting for more than 5% of the entire *C. elegans *genome [19]. Evidence that this GPCR [WormBase: F54D7.3] is orthologous to the human GnRHR1 and Dm-AKHR is supported by the findings that, 1) the next closest potential orthologue to human GnRHR1 (UDP-glucuronosyltransferase; [GenBank: NM_073809]) has considerably less identity (amino acid = 13.8%, E-score = 7.1) to human GnRHR1 than F54D7.3 (amino acid = 22.2%, E-score = 0.45). Likewise, the next closest potential orthologue to Dm-AKHR (hypothetical protein Y116A8B.5; [GenBank: CAA16290]) has less identity (amino acid = 21.8%, E-score = 2e-18) to Dm-AKHR than F54D7.3 (amino acid = 28.7%, E-score = 2e-45); 2) the alignment of F54D7.3 with human GnRHR1 and two other Class A GPCRs, rhodopsin and vasopressin receptor type 1A, showed that the functionally important amino acids of F54D7.3 were significantly more similar to the same sites in human GnRHR (56%; Table 2). Likewise, the alignment of F54D7.3 with Dm-GnRHR also indicated significant similarity between the functionally important amino acids (66%; Table 2), and 3) this GPCR was actively transcribed in the adult worm (Fig. 2C) and its translated protein was localized to the germline, fertilized eggs, intestine, and pharynx (Figs. 3, 4, 5, 6). Likewise, in *Drosophila melanogaster *AKHR was most highly expressed in ovaries, digestive system, brain, tracheae and fat body cells, although immunoreactivity appeared to be more cytoplasmic in nature [20].

Phylogenetic analysis supports the idea that the evolutionary relationships of Ce-GnRHR place it somewhere between the vertebrate GnRHRs and insect AKHRs, though it is more closely allied with AKHRs (Fig. 7; Table 2). The strict consensus tree of select Class A and B GPCRs (Fig. 7) provides minimal bootstrap support for many of the deeper nodes, but three conservative conclusions relevant to our study of Ce-GnRHR may be drawn from the phylogenetic analysis: (1) Ce-GnRHR is more closely related to insect AKHRs than to chordate GnRHRs or corazonin receptors, (2) vertebrate GnRHRs comprise a distinct group of receptors, separate from all other GnRHR-like receptors, including Ce-GnRHR, and, (3) the classification of Ce-GnRHR [F54D7.3] as a GnRHR-like receptor (as opposed to another class of GPCR) is supported.

The Ce-GnRHR ligand has not been identified. Intriguingly, though, we have shown that human GnRH increases both egg laying (17%) and viable offspring (42%) in *C. elegans *(Vadakkadath Meethal et al., 2004 [21] and unpublished data), although it is unclear at this stage whether human GnRH1 binds Ce-GnRHR. GnRHR and GnRH/GnRH-like oligopeptides have been identified in all mammalian and non-mammalian vertebrate species [11,22] studied to date, as well as other vertebrates and invertebrates (octopus, tunicates, lamprey, fish, frogs, etc) [23-25]. While a variety of invertebrate species, including numerous insects and the oyster, *Crassotrea gigas*, have been shown to possess GnRHR orthologues, insects bind a distinct, non-GnRH-like peptide (AKH) [13,24]. In insects, AKHs are secreted from endocrine cells of the corpora cardiaca into the hemolymph [26] and mobilize energy reserve from storages (from fat body) and regulate energy homeostasis [27] by signaling through AKHRs.

Molecular phylogenetic analyses of the past decade have garnered increasingly strong support for the group Ecdysozoa [28,29], the major phyla of which are Arthropoda and Nematoda. Under this phylogenetic hypothesis, it is not unreasonable to expect a nematode GnRHR will bind an AKH-like peptide. Yet, an invertebrate GnRH peptide has been biochemically characterized in the mollusk *Octopus vulgaris*. Given the placement of Ce-GnRHR in the topology of our phylogenetic hypothesis – between the AKHRs and the molluscan GnRHR (oyster), whose ligand is unknown, but may very well be GnRH – another possibility is that Ce-GnRHR is a receptor capable of binding AKH- *and *GnRH-like ligands. A final, equally plausible possibility is that Ce-GnRHR binds an altogether different hormone specific to nematodes, which is supported by the relatively low conservation of ligand binding amino acid residues for both human GnRHR and Dm-AKHR (Table 2).

Ce-GnRHR localized to the nucleus of germline and intestinal cells, as well as to the myofilamant lattice of the pharyngeal musculature (Fig. 5). Although GnRHR1 is traditionally thought of as a plasma membrane receptor [30,11], it is becoming increasingly evident that GnRHR1 can be internalized from the plasma membrane to the nucleus [18]. Indeed, the nuclear localization of GnRHR1 has been reported in rapidly proliferating cells such as pancreatic and breast cancer cells [17,31]. Ligand binding to GnRHR may be the stimulus for the nuclear internalization of the receptor since GnRH has been shown to be rapidly internalized to the nuclear membrane prior to entry into the nucleus [18]. Although we did not detect intense Ce-GnRHR staining in germline and intestinal plasma membranes (Fig. 4), it remains to be determined whether Ce-GnRHR localized to the nucleus (Fig. 6) is from *de novo *receptor synthesized in the cytosol or from Ce-GnRHR translocalization from the plasma membrane. Nevertheless, the localization of Ce-GnRHR to the nucleus provides an exceptional molecular marker of nuclear growth as germ cells progress through the gonadal arm prior to fertilization (Fig. 4).

In vertebrates, GnRH neurons originate from the olfactory placode during organogenesis [32-36]. GnRH secretion from hypothalamic neurons is tightly influenced by environmental conditions [37-40], and this environmental sensing mechanism regulates reproduction [41,42]. Indeed, it has been demonstrated that GnRH1 increases the excitability of olfactory receptor neurons, that the terminal nerve functions to modulate the odorant sensitivity of olfactory receptor neurons and that this signaling is tightly linked to reproduction [43]. The localization of Ce-GnRHR to the germline, pharynx, and intestine (Figs. 4, 5, 6) is suggestive of a role in modulating reproductive function in accordance with environmental conditions. Like the human, the nematode also regulates reproduction dependent upon environmental signals [44] and it is well demonstrated that reproduction in *C. elegans *is strictly controlled by environmental cues such as food and temperature. Under adverse conditions (starvation, high population densities and high temperature), *C. elegans *can enter a reproductively inactive alternative third larval stage called dauer [45,46]. The decision to enter the developmentally arrested dauer larval stage is triggered by a combination of signals from sensory neurons in response to environmental cues [45-47]. Although signaling between olfactory neurons and the reproductive system has been demonstrated in *C. elegans *[41], it is unclear what signaling pathways are involved. It does however suggest the presence of an endocrine system that regulates reproduction in response to environmental conditions.

We propose a model whereby a putative signaling peptide (GnRH, GnRH-like peptide, and/or AKH) in *C. elegans *may be released into the body of the worm from neurons in the head during favorable conditions, where it can then act to signal through Ce-GnRHR located on the pharyngeal musculature, intestine and germ cells (Figs. 4, 5, 6). In this way, this peptide signaling can simultaneously initiate both pharyngeal pumping and reproduction when food is plentiful. Interestingly, the dauer-inducing pheromone detected by sensory neurons in *C. elegans *signals by a complex pathway to the germline, pharynx, intestine, and the ectoderm [46,48]. Indeed, it has recently been shown that octopus GnRH (oct-GnRH) has a contractile effect on the radula retractor muscle which expresses oct-GnRHR [23], and that oct-GnRH mRNA-expressing cell bodies and immunoreactive fibers are present on the superior buccal lobe suggesting that oct-GnRH is involved in feeding behavior generated by contractions of the muscle of the buccal mass [49]. The coupling of food intake to reproduction by such a mechanism would allow for the rapid development and subsequent reproduction of the worm. It is intriguing that Ce-GnRHR is closely related evolutionary with human GnRHR and insect AKHR, involved in regulating reproduction and metabolism, respectively. Ce-GnRHR may provide a molecular link between reproduction and metabolism.

## Conclusion

The sequence similarity, structural organization and localization of Ce-GnRHR provide evidence of an evolutionarily conserved GnRHR orthologue in *C. elegans*. Coupled with the presence of a leucine-rich GPCR (LGR) in *C. elegans *with sequence similarity to vertebrate gonadotropin receptors [9] and the detection of estrogen binding proteins in *C. elegans *[50], these results suggest the existence of an ancestral endocrine system for the regulation of reproduction in *C. elegans*. Whilst our studies are suggestive of a role of Ce-GnRHR in reproduction in *C. elegans*, further studies are required to elucidate the signaling pathways and functional role of this GPCR. Identification of the Ce-GnRHR ligand will provide important insights into evolutionary biology, invertebrate systematics, and the reproductive neuroendocrinology of nematodes. Regardless, our identification of an evolutionarily conserved GnRHR in *C. elegans *opens the way to using this organism as a model system to study reproductive endocrinology.

## Methods

### Nematode strain

The wild type N2 Bristol (Caenorhabditis Genetics Center, National Institutes of Health, National Center for Research Resources, MN) strain was cultured at 22–24°C under standard conditions on *E. coli *[51].

### Materials

GnRHR1 monoclonal antibody (F_1_G_4_) raised against the N-terminus 1–29 amino acids of human GnRHR1 was a kind gift from Dr. Anjali Karande of the Indian Institute of Science, Bangalore, India [52]. Rabbit polyclonal antibodies were raised against C-terminus amino acids 386 to 401 (Ac-GIDKRNHNVQLEIIDFC-OH) of Ce-GnRHR (The New England Peptides Inc., Gardner, MA), a region not found in human GnRHR1. This sequence was chosen based on antigenicity and to limit cross-reactivity with other proteins. The C-terminus included a cysteine for coupling purposes and the N-terminus was blocked by acetylation. Rabbits were immunized with this HPLC-purified antigen (structural confirmation was determined by mass spectrometry) using standard procedures (The New England Peptides Inc., Gardner, MA). Titer levels were monitored periodically in the animals and the animals bled after 60 days. The serum was then affinity purified (The New England Peptides Inc., Gardner, MA) for use in immunodetection assays. Secondary antibodies including goat anti-mouse IgG-HRP (sc-2055; for the GnRHR1 monoclonal antibody), goat anti-rabbit IgG-HRP (sc-2054; for the Ce-GnRHR antibody) as well as the Western blotting luminal reagent were from Santa Cruz Biotechnology, Inc., Santa Cruz, CA.

### Sequence analyses and structural prediction

A BLASTp [53] search was performed against the *C. elegans *genome using human GnRHR1 and GnRHR2 as query sequences. For each query, the hit with the lowest E-value was aligned with human GnRHR1 using ALIGN [54]. Transmembrane regions of Ce-GnRHR were predicted using the following programs: TMHMM V.2.0 [55], DAS-TMfilter [56] and PSIPRED [57]. In order to determine the level of conservation of functionally important amino acid residues, Ce-GnRHR was aligned with human GnRHR1 [GenBank: NP_000397], Dm-AKHR [GenBank: AAC61523], human vasopressin receptor type 1A [GenBank: NP_000697], and human rhodopsin [GenBank: NP_000530]. Homologous FIRs were identified through comparison to a previously reported structural prediction of human GnRHR1 [11]. In pairwise comparisons, FIRs were scored as 'similar' based on the BLOSUM62 substitution matrix – i.e., if the two residues belonged to any one of the following groups of similar amino acids, which have positive scores in the BLOSUM62 matrix: [WYF], [ST], [AS], [RKQ], [NSHD], [DE], [QKE], [YH], or [VIML]. Percent similarity for each type of FIR was calculated using the formula: (identical comparisons + (0.5 * similar comparisons))/total comparisons) * 100. Comparative genomic organization of Ce-GnRHR, human GnRHR1, and Dm-AKHR was deduced from comparison of our sequence alignment with the exon and intron lengths retrieved from GenBank sequence annotations.

### RT-PCR analysis

Total RNA was isolated from 10 adult worms using TRIzol reagent (Invitrogen, Carlsbad, CA) according to manufacturer's instructions. Ce-GnRHR cDNA was synthesized and amplified using the SuperScript III One-Step RT-PCR system (Invitrogen, Carlsbad, CA). Both cDNA synthesis and PCR amplification were carried out using gene specific primers: 5' – GGT AAA AGT TCG ACG GGT GCA - 3' and 5' - GTT ATT TGT TTT GCC GCC GTC A - 3'. PCR product was run on a 4% Metaphor agarose gel (Cambrex Bio Science, Rockland, ME), and DNA was extracted from bands using a QIAquick gel extraction kit (Qiagen, Valencia, CA). Extracted PCR product was cycle sequenced using a BigDye Terminator v3.1 Cycle Sequencing Kit (Applied Biosystems, Madison, WI) and automated sequencing was performed at the University of Wisconsin Biotechnology Center (Madison, WI). To ensure that sequenced PCR products were indeed Ce-GnRHR cDNA and not the result of amplification of residual genomic DNA in the RNA sample, sequenced PCR products were aligned with *C. elegans *cosmid F54D7 [GenBank: AF039712] and checked for the absence of intronic sequence.

### Western immunoblotting

Worms raised in liquid culture were pelleted by centrifugation at 800 *g *for 5 min., washed 3 times with S-basal and then filtered through a Whatman filter paper No. 1 (70 mm) under mild suction in order to remove adherent bacteria. Worms were collected, washed in S-basal and pelleted by centrifugation at 800 *g *for 3 min. Worms and bacteria were collected separately, re-suspended in a small volume of S-basal containing protease inhibitors (1 mM phenyl methane sulfonyl fluoride, 10 μg/ml each of aprotinin and leupeptin, 1 μg/ml of Pepstatin A; Roche Diagnostics, Indianapolis, IN), and the samples then subjected to 4 cycles of sonication at 30 Hz with intermittent cooling. Following protein assay, 40 μg of total nematode and bacterial protein (control) were resuspended in sample buffer (50 mM Tris-HCl, pH 6.8, containing 2% (w/v) SDS, 10% glycerol, 1.25% β-mercaptoethanol and 0.1% bromophenol blue) and separated using polyacrylamide gel electrophoresis (10–20% Tricine gels, Invitrogen, Carlsbad, CA). Following electrophoresis and electrophoretic transfer (Immobilon-P transfer membrane pore size 0.45um, Millipore) membranes were probed with antibodies using standard techniques as previously described [58].

### Immunohistochemistry

Isolated eggs and hermaphrodite worms were washed in M9 buffer prior to mounting on poly-L lysine (100%) coated slides and cover slipped. Following the wicking of residual liquid from the slide with Whatman paper, worms were freeze fractured according to Miller and Shakes [59]. Briefly, slides were frozen on dry ice for 30 min., the cover slip was quickly removed using a razor blade and the slide then immersed in cold methanol followed by cold acetone (5 min. each). Immunostaining was performed as per standard procedures. Briefly, worms were washed in 1% NGS (in TBS) for 10 min., 10% NGS for 30 min. and 1% NGS for 1 min. Slides were incubated with the anti-human GnRHR1 antibody F_1_G_4 _(dilution: 1:250) or with the affinity purified anti-Ce-GnRHR antibody (1:125) overnight at 4°C. Slides were then rinsed in 1%, 10% and 1% NGS for 10 min. each prior to incubation with secondary antibody for 30 min. at room temperature. Slides were washed with 1% NGS three times prior to DAB staining and mounting with Vectashield (Vector Laboratories, Inc. Burlingame, CA). Controls treated with secondary antibody alone were similarly processed. Images were acquired using a Zeiss Axiovert 200 inverted microscope connected to a Fluo Arc light source and an Axio Cam MRC-5 camera. Images were visualized and scaled using Axio Vision 4.0.

For whole-mounted fluorescent immunohistochemistry, hermaphrodite worm cuticles were permeabilized using Tris-Triton buffer with 1% mercaptoethanol and cellular contents fixed according to Finney and Ruvkun [60] as modified by Miller and Shakes [59]. Worms were immunoprobed with GnRHR antibodies and fluorescently tagged secondary antibodies and images captured as described above. To localize nuclei, Hoechst dye (20 μl of 7 μg/μl) was added to the mounting medium and fluorescent staining detected using a DAPI filter [59].

### Phylogenetic analysis

The amino acid sequences of 52 Class A and B GPCRs were retrieved from the GPCR data base [61] including representatives of vertebrate GnRHRs, insect cardioregulatory peptide receptors, corazonin receptors, and AKHRs, and a variety of rhodopsin, vasopressin, and oxytocin receptors from a wide variety of animal species. Sequences were aligned in ClustalX [62] using default gap penalties, and any positions of the initial alignment represented by less than half of the sequences were excised in BioEdit [63]. Excision resulted in a 422 amino acid dataset centered on the seven transmembrane domains and the intervening intra- and extracellular domains. Maximum parsimony analysis (1000 random sequence addition replicates with TBR perturbation) of the dataset was performed in PAUP*4.0b10 [64]. An unrooted, strict consensus tree was constructed from the most parsimonious trees. Bootstrapping, also performed in PAUP, was used to assess the support for relationships represented in the consensus tree: 500 boostrap replicates, 10 random sequence addition each.

## Authors' contributions

SVM, MJG, RJH and SJP designed and coordinated the *C. elegans *experiments, including the immunohistochemistry, western blotting, analysis and interpretation of data. MJG, JYS and SVM performed the sequence analyses and structural predictions. RJH performed the mRNA experiments and phylogenetic analyses. CSA and SVM conceived the study and designed the experiments. SVM, CSA, MJG and RJH wrote the manuscript. All authors read and approved the manuscript.

## Supplementary Material

Additional file 1 **Prediction of transmembrane domains (TMs) in human GnRHR1 and Ce-GnRHR**. The sequence alignment of human GnRHR1 and Ce-GnRHR presented here is taken from Figure 1; Thus for consistency, some sites are represented by gaps in both sequences. Functionally important residues (FIRs) are colored (see legend for classification). Overall, there is 56 % similarity between the FIRs of human GnRHR1 and Ce-GnRHR. TMs for both human GnRHR1 and the Ce-GnRHR were predicted using multiple programs described in Methods and are represented as colored lines above/below the alignment. Each program predicted Ce-GnRHR, like human GnRHR1, to have 7 TMs. Click here for file

Additional file 2 **Expression of the GnRHR1 orthologue in *C. elegans***. Worm (N2) homogenates were immunoblotted with a monoclonal antibody against human GnRHR1. The antibody recognized a 46-kDa band, the calculated molecular weight of the GnRHR1 orthologue. The secondary antibody control did not show immunoreactivity. The molecular weight markers are indicated in kDa on the left. Click here for file

Additional file 3 **Comparison of sequence and genomic organization between Ce-GnRHR and the putative orthologue to human GnRHR2 (orthologue 2)**. (A). Pairwise sequence alignment of Ce-GnRHR and orthologue 2 [GenBank; NP_506566]. The Ce-GnRHR sequence used to raise the anti-Ce-GnRHR antibody is highlighted with a green box. (B). Comparative genomic organization of Ce-GnRHR and orthologue 2. Exon colors in orthologue 2 sequences correspond to homologous regions in the Ce-GnRHR sequence. The gray box delimits the C-terminus portion of Ce-GnRHR absent from orthologue 2. Click here for file

Additional file 4 **Class A and B GPCR dataset used to construct phylogenetic tree (Fig. **7**)**. Sequences are given in FASTA format. Click here for file
