# Supplementary material for: Identification of a gonadotropin-releasing hormone receptor orthologue in Caenorhabditis elegans
Source: BMC Evol Biol. 2006 Nov 29;6:103. doi: 10.1186/1471-2148-6-103 (PMC1762030; doi:10.1186/1471-2148-6-103)
Supplement: Additional file 4 — Class A and B GPCR dataset used to construct phylogenetic tree (Fig. 7). Sequences are given in FASTA format. [file 1471-2148-6-103-S4.pdf]

>sctr_Oryctolagus_cuniculus [EMBL: O46502]
PDLACGVSMNDSSHERQHAYLLKLKVMYTVGYSSSLVMLLVALGILCAFRRLHCTRNYIHMHLFLSFILRALSNFIKDAVLFS
SDDAIHCDAHR-----------------------------------
VGCKLVMVFFQYCIMANYAWLLVEGLYLHSLLVVSFFSERKC----
LQGFVVLGWGSPAMFVTSWAVTRHFLED-----------------------------------
SGCWDINANAAIWWVIRGPVILSILINFILFINILRILTRKLRTQETRGQD--------------------------------
------------MNHYKRLARASTLLLIPLFGVHYIVFVFSPE-GAMEIQL----------
FFELALGSFQGLVVAVLYCFLNGEVQLEVQKKW-------
>ccapr_Drosophila_melanogaster [GenBank: JC7952]
------LNSFYFYETEQFAVLWILFTVIVLGNSAVLFVMFINK-------
NRKSRMNYFIKQLALADLCVGLLNVLTDIIWRIT---ISWRAGN-----------------------------------
LACKAIRFSQVCVTYSSTYVLVAMSIDRYDAITHPMNFSKSWK--
RARHLVAGAWLISALFSLPILVLYEEKLIQGH--------------------PQCWIEL-----GSPIAWQVYM--
SLVSATLFAIPALIISACYAIIVKTIWAKGSIFVPTER--AGFGAAPARRASS--------------------------
RGIIPRAKVKTVKMTLTIVFVFIICWSPYIIFDLLQVFGQIP-H-
SQTNIAIATFIQSLAPLNSAANPLIYCLFSSQVFRTLSRFPPFKWFT-
>caccr_Drosohpila_melanogaster [GenBank: Q868T3]
------LNSFYFYETEQFAVLWILFTVIVLGNSAVLFVMFINK-------
NRKSRMNYFIKQLALADLCVGLLNVLTDIIWRIT---ISWRAGN-----------------------------------
LACKAIRFSQVCVTYSSTYVLVAMSIDRYDAITHPMNFSKSWK--
RARHLVAGAWLISALFSLPILVLYEEKLIQGH--------------------PQCWIEL-----GSPIAWQVYM--
SLVSATLFAIPALIISACYAIIVKTIWAKGSIFVPTER--AGFGAAPARRASS--------------------------
RGIIPRAKVKTVKMTLTIVFVFIICWSPYIIFDLLQVFGQIP-H-
SQTNIAIATFIQSLAPLNSAANPLIYCLFSSQVFRTLSRFPPFKWFT-
>ccapr_receptor_Anopheles_gambiae [GenBank: NP_996297]
-TRGEEINSFYFYETEQFAFLWVLFTVIVLGNSAVLVTLMLNR-------
TRKSRMNFFIKQLAIADLCVGLLNVLTDIIWRIT---VVWRAGN-----------------------------------
AACKAIRFVQVCVTYASTYVLVALSIDRYDAITHPMNFSGCWS--
RARKLVAAAWSFSILFSLPITYFYEERLIQGK--------------------MQCWIDL-----VEAWRWQLYM--
CWVSGSLFVVPALIISACYAVIVRTIWAKGTILGPIDRTHNGMADLATRRASS--------------------------
RGIIPRAKVKTVKMTIVIVIVFVLCWSPYIIFDLLQVFGQIP-A-
TQTNVAIATFIQSLAPLNSAANPLIYCLFSTQVCRMIKRLPPFRWLWS
>oxyr_Homo_sapiens [GenBank: NP_000907]
------------LARVEVAVLCLILLLALSGNACVLLALRTTR-------
QKHSRLFFFMKHLSIADLVVAVFQVLPQLLWDIT---FRFYGPD-----------------------------------
LLCRLVKYLQVVGMFASTYLLLLMSLDRCLAICQPLRSLRR---
RTDRLAVLATWLGCLVASAPQVHIFSLREVADG-------------------VFDCWAVF-----IQPWGPKAYI--
TWITLAVYIVPVIVLAACYGLISFKIWQNLRLKTAAAAAAEAP--EGAAAG--DGGRVA--------------
LARVSSVKLISKAKIRTVKMTFIIVLAFIVCWTPFFFVQMWSVWDAN----
APKEASAFIIVMLLASLNSCCNPWIYMLFTGHLFHELVQRF-------
>oxyr_Bos_taurus[GenBank: NP_776559]
------------LARVEVAVLCLILFLALSGNACVLLALRTTR-------
HKHSRLFFFMKHLSIADLVVAVFQVLPQLLWDIT---FRFYGPD-----------------------------------
LLCRLVKYLQVVGMFASTYLLLLMSLDRCLAICQPLRSLSR---
RTDRLAVLVTWLGCLVASAPQVHIFSLREVADG-------------------VFDCWAVF-----IQPWGPKAYI--
TWITLAVYIVPVIVLATCYGLISFKIWQNLRLKTAAAAAEAAAGAEGEAAD--WAGRAI--------------
LARVSNVKLISKAKIRTVKMTFIVVLAFIVCWTPFFFVQMWSVWDAD----
APKEASPFIIAMLLASLNSCCNPWIYMLFTGHLFQELVQRF-------
>oxyr_Gallus_gallus [GenBank: NP_001026740]
------------MAKVEVTVLCLILFLALSGNLCVLLAIHTTR-------
QKHSRMYFFMKHLSIADLVVAVFQVLPQLIWDIT---FRFYGPD-----------------------------------
FLCRLIKYLQVVGMFASTYMLLLMSLDRCLAICQPLRSLHR---
RADRVSVLLTWLLCLLVSIPQIHIFSLRDVGNG-------------------VYDCWADF-----IQPWGPKAYV--
TWITLMVYIIPVLMLSVCYGLISFKIWQNVKLKTAHGPP------GGQSST--ARGGAA--------------
FARVSSTRLISKAKIRTVKMTFIIVLAFIVCWTPFFFVQMWSVWDTN----
APQEASPFIIAMLLASLNSCCNPWIYMLYTGHLFHDLMRRF-------
>vasr1a_Homo_sapiens [GenBank: NP_000697]
------------LAKLEIAVLAVTFAVAVLGNSSVLLALHRTP-------
RKTSRMHLFIRHLSLADLAVAFFQVLPQMCWDIT---YRFRGPD-----------------------------------

WLCRVVKHLQVFGMFASAYMLVVMTADRYIAVCHPLKTLQQPA-
RRSRLMIAAAWVLSFVLSTPQYFVFSMIEVNNVT-----------------KARDCWATF-----IQPWGSRAYV--
TWMTGGIFVAPVVILGTCYGFICYNIWCNVRGKTAS-RQSKGAE--QAGVA-FQKGFLL--------------
APCVSSVKSISRAKIRTVKMTFVIVTAYIVCWAPFFIIQMWSVWDPMSVW-
TESENPTITITALLGSLNSCCNPWIYMFFSGHLLQDCVQSF-------
>vasr1a_Tribolium_castaneum [GenBank: XP_967376]
------------LARVEVATLAIIFLVTVIGNSTVLLALWTRRRYAG--
RKKLSRMYFFILHLSIADLITAFLSVLPQLAWDIT---YRFYGGF-----------------------------------
LLCKVVKYGQTLGPYLSSYVLMATAIDRHQAICYPLTYCSWTS-
RRSKVMVYLAWVASLAFCIPQLTIFTYTSVGED-------------------EYDCWATF-----QEPWGKRAYV--
TWYSISVFMVPLVVLIFTYTSICIEIWQSSESSLRPRSS-------QKSAP----GKR---------------------
TPLISRAKINTVKQTIAVIVMYIACSTPFILAQLWATWDPQ-------------------------
SPFIDVEFKKVAQEDYIP---------
>vasr2_Homo_sapiens [GenBank: NP_000045]
------------LARAELALLSIVFVAVALSNGLVLAALARRGR-----
RGHWAPIHVFIGHLCLADLAVALFQVLPQLAWKAT---DRFRGPD-----------------------------------
ALCRAVKYLQMVGMYASSYMILAMTLDRHRAICRPMLAYRHGSGAHWNRPVLVAWAFSLLLSLPQLFIFAQRNVEGGS-----
------------GVTDCWACF-----AEPWGRRTYV--
TWIALMVFVAPTLGIAACQVLIFREIHASLVPGPSERPGGRRR---GRRTGSPGEG------------------------
AHVSAAVAKTVRMTLVIVVVYVLCWAPFFLVQLWAAWDPE----
APLEGAPFVLLMLLASLNSCTNPWIYASFSSSVSSELRSLL-------
>gnrhr2_Homo_sapiens [GenBank: Q96P88]
-VEGSELPTFSAAAKVRVGVTIVLFVSSAGGNLAVLWSVTRREPSQ----
LRPSPVRRLFIHLAAADLLVTFVVMPLDATWNIT---VQWLAVD-----------------------------------
IACRTLMFLKLMATYSAAFLPVVIGLDRQAAVLNPLGSRSGV--RK---
LLGAAWGLSFLLAFPQLFLFHTVHXAGPV-----------------PFTQCVTKG---SFKAQWQETTYN--
LFTFCCLFLLPLTAMAICYSRIVLSVSRPQTRKG---SHAPAGEFALPRSFDN-----------------------------
CPRVRLRALRLALLILLTFILCWTPYYLLGMWYWFSPTMLT--
EVPPSLSHILFLLGLLNAPLDPLLYGAFTLGCRR-------------
>gnrhr2_Sus_scrofa [GenBank: AAS68622]
-VESSELPTFSVAAKVRVGVTVVLFVSSAGGNLAVLWSVTRPQPSQ----
LRPSPVRRLFAHLAAADLLVTFVVMPLDASWNIT---VQWLAGD-----------------------------------
IACRTLMFLKLMAMYAAAFLPVVIGLDRQAAVLHPLGPRSGG--RK---
LLGAAWLLSFLLALPQLFLFHTVRRAGPV-----------------PFTQCVTKG---SFKAQWQETTYN--
LFTFCCLFLLPLTVMTICYSRIVLSVSSPRTRKG---NDAPAGEFTLRRSLDN-----------------------------
RPRVRLRALRLALLVLLTFVLCWTPYYLLGLWYWFSPTMLS--
EVPPSLSHILFLFGLLNAPLDPLLYGAFTLGCRR-------------
>gnrhr1_Rana [GenBank: AAO50198]
-HTHFQLPTFSPAAKARVIITFVIFTLSATCNLAALWSAARTS--R----
KKRSHVRILILNLTTADLLVTFIVMPLDAIWNIT---VQWHAGD-----------------------------------
IACRILMFLKLLSMYSCAFVTVVISVDRQSAILNPLAINDAK--
KKNKIMLSVAWLMSAVLSLPQLFLFHTVTITEPH-----------------NFTQCTTRG---SFQQHWQETVYN--
MVSFVCLFLLPLLIMICCYSRILLEISKRMSK-----GTLSSKEVYLRCSKNN-----------------------------
IPKARMRTLKMSVVIVSSFIICWTPYFLLGLWYWFYPEIME-
EKVSQSTTHILFIFGLVNACLDPITYGLFTIHFRKSLQRYC-------
>gnrhr_Typhlonectes_natans [GenBank: AAD49750]
-------PTFSTAAKVRVTITFVLFISSACFNIIALWTITQK---Y----
KKRSHVRILISNLAVADLLVTFIVMPLDAIWNIT---VQWYAGD-----------------------------------
LVCRVLMFLKLVAMYASAFVTVVISLDRQSAILNPLGIGDAK--
KKNKIMLCVAWVLSVLLAVPQLFVFHAVSPSQSE-----------------YFIQCATVG---SFQGHWQETLYN--
MFTFSCLFLLPLLIMVLCYSRILIEISRKMKK-----ACVSSKEVHLRRSSNN-----------------------------
IPKARLRTLKMSIVIVLTFIVCWTPYYLLGIWYWFSPEMLTRERVPPSLSHILFLFGLFNACLDPLIYGLFTI----------
----
>gnrhr3_Oryzias_latipes [GenBank: BAC97833]
--DALQLPTFSTAAKVRVIITFTLCAVSAVCNLLVLWAAGKG---G----
KRKSHVRILIMNLTVADLLVTFIVMPVDAAWNIT---VQWQAGD-----------------------------------
LACRLLMFMKLVAMYSCAFVTVVISLDRQSAILNPLGISEAK--
RKSKIMLTVAWTTSVILSLPQMFIFHNVTISVPE-----------------NFTQCTTHG---SFVQRWQETLYN--
MFTFVCLFLLPLVIMIFCYTRILVEISSRIAR-----TNMVSRDIHLRRSHNN-----------------------------

IPKARMRTLKMSIVIVTSFIICWTPYYLLGLWYWLFPEKME-
ETVSHSLTHMLFIFGLFNACLDPITYGLFTIHLRQGAKR---------
>gnrhr_Ciona_intestinalis [GenBank: NP_001027765]
-VLIQTWFQFDTLHLVRVLVTWLLFCLSMAGNMFVLWSLRGS-----------
KSRHFIMFHLALSNLVYTIFVMPSDAVWNTT---MEWLAGD-----------------------------------
VMCRLCQMMKQFGMYASSFMVVVIGADRVTGILSPLPCHSQR--
KRGYYMVATAWISSLICCLPAGFIFSVASIPTCEG---------------IPIYQCIDFN---VLQDVSLLRPYY--
FFTMCMSFLLPLICTLVSYSLIVCEISNMKER--------DRVLMGRRHSVNTAS---------------------------
IQRAKNRTILMRTLITLTFLVCWGPYYGKGIYDWFIRYEDH--
TPPDAWDTVMYVVMYLNPVLHPIVFGVFLKEIRGKFKQRLN------
>akhr_Tribolium_castaneum [GenBank: ABE0225]
--KLPISMRFNEGHQLSIIVYSILMVFSAIANTTVLVLIVKRRR------
KTPSRINTMLMHLAIADLLVTFLMMPLEIGWAST---VSWYAGD-----------------------------------
AMCRIMMFFRMFGLYLSSFILVCISVDRFYAVLKPLYLRALD--
RRDKFMLLGAWLGATLCSIPQMVVFHVESHPNIT-----------------WYQQCVTYN---VFPTYAHELTYL--
LFGMVMMYALPLAVIIFSYASILLEIRRRTR--------NPYGDSVTRSSLAF-----------------------------
LGKAKVRTLKMTIIIVLVFFVCWTPYYVMCIWYWLDR--
ESAKNVDQRIQKALFLFACTNSCMNPVVYGVFNIRA---------------
>akhr_Anopheles_gambiae [GenBank: ABD60146]
-YEMPIDMRFNSGHILSIMVYTTLMVFSATGNLTVLSILAQRKV------
RASSRINIMLAHLAIADLLVTFLMMPLEIGWAYT---VRWTAGD-----------------------------------
LMCRVMAFFRTFGLYLSSFILICISVDRYFAVLKP--LKVHE--
HRAVLMIAAAWIMSGLCSLPQAFIFHLEGHPNIT-----------------GYQQCVTYH---YFEEEIYQIIYN--
VLVMCLMYTFPLIVILYCYGSIYYEIFSRTN--------PRNLESFRRSSIDV-----------------------------
LGRAKRKTLRMTIMIVIVFVVCWTPYYVMSLWYWLDK--
ESTKNVDQRIQKGLFLFASTNSCMNPVVYGVFNVRK---------------
>akhr_Periplaneta_americana [GenBank: ABB20590]
---MTDDMKFNDGHRMSIITYSILMVVSVAGNSTVLITILKRGR------
TLRYGNNYMFMHLAIADLLVTFLMMPLEIAWNIT---VSWKGGD-----------------------------------
LMCRIMLFFRTFGLFLSSFVIVCISLDRCVAILRPMSKKLLNVARRGKLMLTVAWILATLCSLPQAVIFHVEPHPNVT-----
------------WYEQCVSFN---FFSTKMHEFTYR--VLGMVMMYGLPLIVIVISYACILGEIIRRYQ--------
LSPDDSFRRSSLVF-----------------------------LNRARNRTLKMAIIIFVVFFICWTPYYVMCLWYWIDE--
RSAETVDHRVQKALFLFASTNSCMNPIVYGYFNFRLGRGSGYGATGGRVGQ
>akhr_Bombyx_mori [GenBank: NP_001037049]
--ELPLEMRFNYSHMVSMTVYSVLMVISATGNLTVLYQLVRRRR--A---
KRASRLDILLMHLAVADLMVTFLMMPLEIAWAGT---VQWFAGD-----------------------------------
LMCRVMMFTRTFGLYLSSFVLICIAVDRYYAILKPLNVTWEATVRRA---
IIVAWVCAGLASLPQSFIFHVEEHPEVK-----------------GYNQCVSYG---SLPTEKHEFAYF--
LVNMILMYVIPLVSTLYCSCAALFEIIRRAN--------TAN-DKMRRSGIGL-----------------------------
LGRARARTLKMTVTIVLVFFTCWSPYYCYCLWYWIDK--
ESIKNLDPALQKAMWLFSCTNSCANPIVYGVFNRNRWNWRAGKFQNGRCRS
>gnrhr_Crassostrea_gigas [GenBank: CAI64587]
-------LKFSDNYAHSIISLGVVFLIGALGNIASFVTLLCSKN-------
RKSPTNFFLLHLSSADLLVMFVIPVGEMIHNAT---IAWLGGN-----------------------------------
FLCKLYHFLWNFGQYVATFLLCCISIDRYLAFVFPLRSLTQS-
PRTRSFMAATAWVLSAILSAPESVIFHVETHPKYR-----------------TFRQCVTFN---FFPSHNHELAYN--
LFNLITLYALPLLIITTSYSLILWEISKKTKQCKEETKCLSTRSRLRRSSVGN-----------------------------
MERARIRTLKMTLVIVSVFVICWTPYFVLSAWWWFDS--
DSASQLDPKVQRGLFLFAVSNSCMDPIVYGMFTT-----------------
>gnrhr1_Caenorhabditis_briggsae [GenBank: CAE60355]
-------IAIKSSVISIVLTYLALFILAFIGNVTMFMILCRNQL------
VKVRRVHSLLLHMNIAHLLVTLVVMPKEILHNYM---VAWFAGD-----------------------------------
IMCRVCKFFDVFGISLSMNVLICITLDRFYSIFFPLYAMRAR--
KSVQRMVSIAWIVSLITSAPQLYLFKTAEHPCFA-----------------WYTQCVSLN---
FIGELSNDVVFYFSIINIIQVYIAPLIVTVICYSLILWKISRKTKLVGEKESEKSSELLLRRNGQNN----------------
-------------LEKARSRTLKMTFVIVLAFISCWTPYSILMFLHFLR----
RTDWIPKDIRKFIYAFAVLNSAISPYLYGYFSF-----------------
>gnrhr1_Caenorhabditis_elegans [GenBank: NP_491453]
-------VTVNDSVLSIVFTYLALFILAFVGNVTMFLILCRNQL------

VKVRRVHSLLLHMNIAHLLVTLVVMPKEILHNYM---VAWFAGD-----------------------------------
VMCRICKFFDVFAISLSMNVLICITLDRFYSIFFPLYAMRAR--
KSVQRMVSFAWTISFVTSAPQLYLFKTATHPCFD-----------------WYTQCVSKN---
FIGELSNDVVFFFSIVNIIQVYIAPLFVTVVCYSLILWRISRKSKLVGEKESEKSSELLLRRNGQNN----------------
-------------LEKAKSRTLKMTFVIVLAFIFCWTPYSILMFLHFLR----
HTDWIPKDIRKFIYAFAVLNSAISPYLYGYFSF-----------------
>corr_Anopheles [GenBank: AAQ67361]
LSCYEHAPTLSKSGVIRVIVLSAMAIVSLLGNVATMWNIQKNRKSRRVTRHNWSAIYSLIFHLSIADVLVTGFCLIGEAAWYY
T---VDWVAGN-----------------------------------
LFCKLFKLCQMFSLYLSTYVLVLVGVDRWVAVKYPMKSLNTA--
RRCHRFLFVAYLLSFLLSTPQWMIFRVAKGPFVE-----------------DFYQCVTHG---FYTDRWQEQLYT--
TFTLVFMFIIPLLILIGTYLSTFMTISSSEKIFRIDTSAVDRTTYYRRSDTNRQ--------------------------
RLIHKAKMKSLRISVVIVVAFVVCWTPYYIMMLIFMFLN---
PTERFGEDLQSGIFFFGMSNSLINPLIYGAFHLVPIRQRRNQYNQHVREG
>corr_Manduca_sexta [GenBank: AAR14318]
-SCLEHAPILTKSTVIRASVLSAMAILSFFGNLATIISIQRGKRGRGRARPSWTAIYSLIFQLSIADLLVTIFCIAGEAAWSF
T---VQWYAGN-----------------------------------
IACKIFKFLQMFALYQSTFILVLIGVDRWLAVKYPMKSMATA--
TRSGRLVVIAWVLSVVLSIPQTVVFRVAKGPFVE-----------------EFYQCVTHG---FYTERWQEQAYT--
TLSLVFMFVLPLVILISTYVSTVRTIARSEKVFKP---EVRRQEKYFTPDMNRR--------------------------
RLIDRAKMKSLRMSVVIVAAFLVWWAPYYVMMIIFTFLN---
PDKDQSEELLSGIFFFGMSNSLVNPVIYGAFHLWPKKKRSHRHSDRESGG
>corr_Drosophila_melanogaster [GenBank: JC7896]
EHVMDHAPQLSRSGLLKVYVLAVMALFSLLGNLLTIWNIYKTRISRRNSRHTWSAIYSLMFHLSIADVLVTWFCIIGEAAWCY
T---VQWLANE-----------------------------------
LTCKLVKLFQMFSLYLSTYVLVLIGVDRWIAVKYPMKSLNMA--
KRCHRLLGGTYILSLVLSLPQFFIFHVARGPFVE-----------------EFYQCVTHG---FYTADWQEQMYA--
TFTLVFTFLLPLCILFGTYMSTFRTISSSEKMFQG-SKLANYSTAKLPTQTNRQ--------------------------
RLIHKAKMKSLRISVVIIIAFLICWTPYYVMMIMFMFLN---
PDKRIGDDLQDAIFFFGMSNSLVNPLIYGAFHLCPGKGGKSSGGGGNNNA
>gnrhr2_Caenorhabditis_briggsae [GenBank: CAE60908]
-------LGYITSDYAQMLIYGIFVLIGLPVNISTLIYMLKRYR------
HAKSFLLLLHINLNISDILVLGLYVPGLIGWLVT---LEWRGGE-----------------------------------
YLCKFMRFVDAFVFAISSNIMVCIALYRLSALRYPLWVNAVG-
HSRVPRMLVLAWSLAVVTMLPQMFVWNEVQFNHITQCVT-------------VWTEIINKEHALSESELRNMKLYS--
IQNAIIIFYVPLMILVACYVLILKDIYKTLNTDTECSSAAYLSEMSSSKTGGKATAHKKEQESFVTLTTRTV---RG--
QEKFRRAKVRSLRITLLLILTYAVTWLPYNLLSWWMVLHFDS---
YKENLDSNYILNSLVVLNSVINPFIYGRCQG-----------------
>gnrhr2_Caenorhabditis_elegans [GenBank: CAB02736]
-------LGHVPSDYAQMLLYGIFALIGLPVNISTLIYMLKRYR------
HAKSFLLLLHINLNISDILVLGLYVPGLIGWLVT---LEWRGGA-----------------------------------
YLCKFMRFVDAFVFAISSNIMVCIALYRLSALRYPLWVNAVG-
HSRVPRMLILAWALAVVTMLPQMLVWNEVQFSNITQCVT-------------VWTEIINKREKLSESELRNMKLYS--
IQNATIIFYIPLMILVACYVLILKDIYKTLNTDTECSSAAYLSEMSSSKTGGKAALHKKDQESFVTLTTRTV---RG--
QEKFRRAKVRSLRITLLLILTYAVTWLPYNLLSWWMVLHWDS---
YRENLDSNYILNSLVVLNSVINPFIYGRCQG-----------------
>rhodlk_Anopheles_gambiae [GenBank: EAA01405]
----QFPPMNPLWHSILGFAIFMLGMISMTGNGCVMYIFTNTKS-------
LRTPSNLLVVNLAFSDFFMMFTMGPPMVINCWH---ETWTFGP-----------------------------------
FACELYAMLGSLFGCASIWTMTMIAFDRYNVIVKGLAGKPMTN-
NGALLRILGVWVFALFWTLAPLFGWNRYVPEGNMT-------------------ACGTDY--LTQT--WLSRSYI--
IIYAIFVYWLPLLTIIYSYTFILKAVSAHEKNMREQAKKMNVASLRTQEAQNT------------------------------
-
STEMKLAKVALVTISLWF---

>rhodlk_Culex_pipens [GenBank: AAP56248]
----QFPPMNPLWHSILGFAIFCLGMVSMIGNGCVISIFTSTPS-------
LKAPSNLLVVNLAFSDFLMMFTMGPPMVINCWH---QTWVFGP-----------------------------------
FACELYACLGSLFGCTSIWTMTLIAFDRYNVIMKGLAAKPMTN-

SGAKIKILGVWAFSLFWTLAPFFGWNRYVPEGNMT-------------------ACGTDY--LTQT--WLSRSYI--
LIYAIFVYWLPLLTIIYSYTFILKAVSAHEEQMREQAKKMNVASLRSSEAQQT------------------------------
-SAEIKLAKVALVTISLWFMARTPYLVINFTGIFKAAP-----
ISPLATIWGSLFAKANAVYNPIVYGIGHPKYRA-------------
>rhodlk_Rana_castesbeiana [GenBank: BAA76864]
----QSHLGTPGMFMSMSAFMLFTIIFGFPLNVLTVICTIKY-------KKLRSHLNYILVNLAVANLIVICFGS-
TTAFYSFSQ--MYFALGT-----------------------------------
LACKIEGFTATLGGMVSLWSLAVVAFERFLVICKPMGSFTFR-
ENHAILGCIFTWVIGLVAASPPLLGWSRYIPEGLQCS-------------------CGPDW--YTVNNKWNNESYV--
IFIFCFCFGFPLAVIVFSYGRLLLTLHAVAKQ--------------QEQSAST------------------------------
QKAEREVTRMVIMMIAGFLVCWLPYASFALWAVTHRG----
ETFDLRMASIPSVFSKASTVYNPFIYIFMNRQFRS-------------
>rhodlk_Homo_sapiens [GenBank: AAX37093]
------AFLPLGLKVTIVGLYLAVCVGGLLGNCLVMYVILRHTK-------
MKTATNIYIFNLALADTLVLLTLPFQGTDILLG----FWPFGN-----------------------------------
ALCKTVIAIDYYNMFTSTFTLTAMSVDRYVAICHPIRALDVRTSSKAQAVNVAIWALASVVGVPVAIMGSAQVEDEEI-----
---------------ECLVEIP---TPQDYWGPVFA--ICIFLFSFIVPVLVISVCYSLMIRRLR---------------
GVRLLSGSREK------------------------------DRNLRRITRLVLVVVAVFVGCWTPVQVFVLAQGLGVQ--
PSSETAVAILRFCTALGYVNSCLNPILYAFLDENFKACFR----------
>vasr1a_Rattus_norvegicus [EMBL: Q5M8D1]
------------LAKLEIAVLAVIFVVAVLGNSSVLLALHRTP-------
RKTSRMHLFIRHLSLADLAVAFFQVLPQLCWDIT---YRFRGPD-----------------------------------
WLCRVVKHLQVFAMFASAYMLVVMTADRYIAVCHPLKTLQQPA-
RRSRLMIATSWVLSFILSTPQYFIFSVIEIEVNNG---------------TKTQDCWATF-----IQPWGTRAYV--
TWMTSGVFVAPVVVLGTCYGFICYHIWRNIRGKTASSRHSKGDKGSGEAVGPFHKGLLV--------------
TPCVSSVKSISRAKIRTVKMTFVIVSAYILCWAPFFIVQMWSVWDENFIW-
TDSENPSITITALLASLNSCCNPWIYMFFSGHLLQDCVQSF-------
>akhr_Apis_mellifera [GenBank: NP_001035354]
---LPIDMRFNEGHIVSIVFYSVLMIISAIGNTTVLILITCRKR------
VSKSRIHIMLMHLAIADLLVTFLMMPLEIGWAIT---VSWKAGD-----------------------------------
VMCRIMAFFRMFGLYLSSFVLVCISMDRYYAVIKPLQLWDVD--
KRGKIMLSFAWIGSVVCSLPQTIVFHLETHPNVT-----------------WYSQCVTFN---AFPTYTHEITYS--
LFGMIMMYWFPLVVIIYTYTSILLEIRRRSK--------KSEDDKIRRSSIGF-----------------------------
LTRAKIRTLKMTVIIIAVFFICWTPYYVMSLWYWIDR--
NSAYKIDQRIQKGLFLFACTNSCMNPIVYGAFNIRD---------------
>vasr1a_Microtus ochrogaster [GenBank: AAD02821]
------------LAKLEIAVLAVIFVVAVLGNSSVLLALHRTP-------
RKTSRMHLFIRHLSLADLAVAFFQVLPQLCWDIT---YRFRGPD-----------------------------------
WLCRVVKHLQVFAMFASAYMLVVMTADRYIAVCHPLKTLQQPT-
RRSRLMIAASWVLSFLLSTPQYFIFSMIEIEVNNG---------------TKTQDCWATF-----IQPWGTRAYV--
TWMTSGVFVVPVVILGTCYGFICYHIWRNVRGKTAS-RQSKG---SGEDVAPFHKGLLV--------------
TPCVSSVKTISRAKIRTVKMTFVIVTAYILCWAPFFIVQMWSVWDDNFIW-
TDSENPSITITALLASLNSCCNPWIYMFFSGHLLQDCVQSF-------
>ghrhr_homosapiens [EMBL: Q02643]
YPVACPVPLELLAEE--
ESYFSTVKIIYTVGHSISIVALFVAITILVALRRLHCPRNYVHTQLFTTFILKAGAVFLKDAALFHSDDTDHCSFST------
-----------------------------VLCKVSVAASHFATMTNFSWLLAEAVYLNCLLASTSPSSRRA----
FWWLVLAGWGLPVLFTGTWVSCKLAFED-----------------------------------
IACWDLDDTSPYWWIIKGPIVLSVGVNFGLFLNIIRILVRKLEPAQGSLHT--------------------------------
------------QSQYWRLSKSTLFLIPLFGIHYIIFNFLPDNAGLGIRL-----------
PLELGLGSFQGFIVAILYCFLNQEVRTEISR---------
>ghrhr_Gallus_gallus [EMBL: Q309X7]
YHIACPVEDEIPLEE--
QSYFSTIKIIYTVGYSLSITSLIIAVTVLMAFRRLRCPRNYIHIQLFFTFILKAIAIFIKDSVLFQEEDIDHCSFST------
-----------------------------TECKISVVFCHYFMMTNFIWLLVEALYLNCLLLSSLSHGRRY----
FWWLVLFGWGFPTLFTFIWILAKFYFED-----------------------------------
TACWDINQDSPYWWLIKGPIIISVGVNFVLFINIIRILLKKLDPRQINFNN--------------------------------
------------SSQYRRLSRSTLLLIPLFGTHYIVFNFLPEYTSLGIRL-----------
YLELCIGSFQGFIVALLYCFLNQEVQTEIGR---------

```
>sctr_Homo_sapiens [EMBL: Q53T00]
PNLACGVNVNDSSNEKRHSYLLKLKVMYTVGYSSSLVMLLVALGILCAFRRLHCTRNYIHMHLFVSFILRALSNFIKDAVLFS
SDDVTYCDAHR-----------------------------------
AGCKLVMVLFQYCIMANYSWLLVEGLYLHTLLAISFFSERKY----
LQGFVAFGWGSPAIFVALWAIARHFLED-----------------------------------
VGCWDINANASIWWIIRGPVILSILINFILFINILRILMRKLRTQETRGNE--------------------------------
------------VSHYKRLARSTLLLIPLFGIHYIVFAFSPE-DAMEIQL-----------
FFELALGSFQGLVVAVLYCFLNGEVQLEVQKKW-------
>calcr_Cavia_porcellus [EMBL: O08893]
NRTWSNYTLCNAFTPEKLQNAYVLYYLAIVGHSMSIITLVVSLGIFVYFRSLGCQRVTLHKNMFLTYILNSMIIIIHLVEVVP
NGELVRKDP-------------------------------------
VSCKILHFFHQYMMACNYFWMLCEGIYLHTLIVVSVFNEAKH----
LRWYYLLGWGFPLVPTTIHAITRALYFN-----------------------------------DNCW-
ISVDTHLLYIIHGPVMVALVVNFFFLLNIVRVLVTKMR--
ETHEAE--------------------------------------------
SYMYLKAVKATMILVPLLGIQFVVFPWRPSNKVLGKIYD----------
YFMHSLIHFQGFFVATIYCFCNNEVQTTLKRQWAQFKIQW
>calcr_Mus_musculus [EMBL: Q60755]
NRTWSNYTLCNAFTSEKLQNAYVLYYLALVGHSLSIAALVASMLIFWIFKNLSCQRVTLHKHMFLTYILNSIIIIIHLVEVVP
NGDLVRRDPMHIFHHNTHMWTMQWELSPPLPLCAHEGKMDPHASEVISCKVLHFLHQYMMSCNYFWMLCEGIYLHTLIVMAVF
TDEQR----LRWYYLLGWGFPIVPTIIHAITRALYYN-----------------------------------DNCW-
LSAETHLLYIIHGPVMVALVVNFFFLLNIVRVLVTKMR--
QTHEAE--------------------------------------------
SYMYLKAVKATMVLVPLLGIQFVVFPWRPSNKVLGKIYD----------
YLMHSLIHFQGFFVATIYCFCNHEVQVTLKRQWTQFKIQW
>rhod_Camponotus_abdominalis [EMBL: Q17292]
----QFPPMNPLWHALLGFVIGVLGVISVIGNGMVIYIFTTTKS-------
LRTPSNLLVVNLAISDFLMMLCMSPAMVINCYY---ETWVLGP-----------------------------------
LFCELYGLAGSLFGCASIWTMTMIAFDRYNVIVKGLSAKPMTI-
NGALIRILTIWFFTLAWTIAPMFGWNRYVPEGNMT-------------------ACGTDY--LTKD--LFSRSYI--
LIYSIFVYFTPLFLIIYSYFFIIQAVAAHEKNMREQAKKMNVASLRSAENQST------------------------------
-SAECKLAKVALMTISLWFMAWTPYLVINYSGIFETTK-----
ISPLFTIWGSLFAKANAVYNPIVYGISHPKYRA-------------
>rhod_Procambarus clarkii [EMBL: P35356]
----QYPPMNPMMYPLLLIFMLFTGILCLAGNFVTIWVFMNTKS-------
LRTPANLLVVNLAMSDFLMMFTMFPPMMVTCYY---HTWTLGP-----------------------------------
TFCQVYAFLGNLCGCASIWTMVFITFDRYNVIVKGVAGEPLST-
KKASLWILTIWVLSITWCIAPFFGWNRYVPEGNLT-------------------GCGTDY--LSED--ILSRSYL--
YDYSTWVYYLPLLP-
IYCYVSIIKAVAAHEKGMRDQAKKMGIKSLRNEEAQKT-------------------------------
SAECRLAKIAMTTVALWFIAWTPYLLINWVGMFARSY-----
LSPVYTIWGYVFAKANAVYNPIVYAISHPKYRA-------------
>rhod_Alligator_mississippiensis [EMBL: P52202]
----QYYLAEPWKYSALAAYMFMLIILGFPINFLTLYVTVQH-------KKLRSPLNYILLNLAVADLFMV-
LGGFTTTLYTSMN--GYFVFGV-----------------------------------
TGCYFEGFFATLGGEVALWCLVVLAIERYIVVCKPMSNFRFG-
ENHAIMGVVFTWIMALTCAAPPLVGWSRYIPEGMQCS-------------------CGVDY--YTLKPEVNNESFV--
IYMFVVHFAIPLAVIFFCYGRLVCTVKEAAAQ--------------QQESATT------------------------------
QKAEKEVTRMVIIMVVSFLICWVPYASVAFYIFSNQG----
SDFGPVFMTIPAFFAKSSAIYNPVIYIVMNKQFRN-------------
>rhod_Astyanax_fasciatus [EMBL: P41590]
----QYYLAPPWAYACLAAYMFFLILVGFPVNFLTLYVTIEH-------KKLRTPLNYILLNLAVADLFMV-
FGGFTTTMYTSLN--GYFVFGR-----------------------------------
LGCNLEGFFATFGGINSLWCLVVLSIERWVVVCKPMSNFRFG-
ENHAIMGVAFTWFMALACTVPPLVGWSRYIPEGMQCS-------------------CGIDY--YTRAEGFNNESFV--
IYMFVVHFLTPLFVITFCYGRLVCTVKEAAAQ--------------QQESETT------------------------------
QRAEREVTRMVILMFIAYLVCWLPYASVSWWIFTNQG----
SEFGPIFMTVPAFFAKSSSIYNPVIYICLNKQFRH-------------
>rhod_Caluromys_philander [EMBL: Q6W3E1]
```

----QYYLAEPWQFSCLAAYMFMLIVLGFPINFLTLYVTIQH-------KKLRTPLNYILLNLAIADLFMV-
FGGFTTTLYTSLH--GYFVFGP-----------------------------------
TGCDLEGFFATLGGEIALWSLVVLAIERYIVVCKPMSNFRFG-
ENHAIMGVAFTWVMALACAAPPLVGWSRYIPEGMQCS-------------------CGIDY--YTLKPEVNNESFV--
IYMFVVHFTIPMVVIFFCYGQLVFTVKEAAAQ--------------QQESATT------------------------------
QKAEKEVTRMVIIMVIAFLICWLPYAGVAFYIFTHQG----
SNFGPILMTLPAFFAKTSAVYNPVIYIMLNKQFRT-------------
>gnrhr1_Cavia_porcellus [EMBL: Q8CH60]
---QGALPTLTLSGKIRVTVTFFLFLVSTTLNASFLLKLQKWTQKKEKG-
KKLSRMKVLLKHLTLANLLETLIVMPLDGMWNIT---VQWYAGE-----------------------------------
LLCKILSYLKLFSMYAPAFMMVVISLDRSMAITRPLPVQSNR--
KLEQSMTGLAWGLSSVLAGPQLYIFKMIHLENGPGQ-------------TEVFSQCVTHC---SFPQWWHQAFYN--
FFTFICLFIIPLLIMLICNAKIIFTLTQVLQQ--------DSNKLQLNQSKNN-----------------------------
IPRARLRTLKMTVAFAASFIVCWTPYYVLGLWYWFDPGMLH--
RMSEPVNHFFFLFAFLNPCFDPLIYGYFSL-----------------
>gnrhr1_Mus_musculus [EMBL: Q01776]
---QGKLPTLTVSGKIRVTVTFFLFLLSTAFNASFLLKLQKWTQKRKKG-
KKLSRMKVLLKHLTLANLLETLIVMPLDGMWNIT---VQWYAGE-----------------------------------
FLCKVLSYLKLFSMYAPAFMMVVISLDRSLAITQPLAVQSNS--
KLEQSMISLAWILSIVFAGPQLYIFRMIYLADGSG--------------PTVFSQCVTHC---SFPQWWHQAFYN--
FFTFGCLFIIPLLIMLICNAKIIFALTRVLHQ--------DPRKLQLNQSKNN-----------------------------
IPRARLRTLKMTVAFATSFVVCWTPYYVLGIWYWFDPEMLN--
RVSEPVNHFFFLFAFLNPCFDPLIYGYFSL-----------------
>gnrhr1_Trichosurus_vulpecula [EMBL: Q9TTI8]
---HRDLPTLTLSGKIRVMVTFFLFLVSTAFNASFLMKLQRQTQKKEEV-
KKLTRMKVLLKHLTLANLLETVIVMPLDGIWNVT---VQWYAGE-----------------------------------
FLCKALSYLKLFSMYAPAFMMVVISLDRFLAITRPLAVKSNT--
KVGQSLIAVAWFLSIVLAGPQLYIFRMIYVEDISGQ-------------TGNFSQCVTHC---SFPEWWQEAFYN--
LLTFSCLFIGPLLIMLVCNAKIIFTLTQVLHQ--------DPHELQLNRSKNN-----------------------------
IPRARLRTLKMTVAFATLFTICWTPYYVLGIWYWFDPEMLN--
RVSDPVNHFFFLFGLLNPCFDPLIYGYFSL-----------------
>gnrhrII_Callithrix_jacchus [EMBL: Q95MG6]
-VEGSELPTFSTAAKVRVGVTIVLFVSSAGGNLAVLWSVTRPQPSQ----
LRPSPVRRLFAHLAAADLLVTFVVMPLDATWNIT---VQWLAGD-----------------------------------
IACRTLMFLKLMAMYAAAFLPVVIGLDRQAAVLNPLGSRSGV--RK---
LLGAAWGLSFLLALPQLFLFHTVHRAGPV-----------------PFTQCATKG---SFKARWQETTYN--
LFTFCCLFLLPLTAMAICYSRIVLGVSSPRTRKG---SHAPAGEFALRRSFDN-----------------------------
RPRVRLRALRLALLVLLTFILCWTPYYLLGLWYWFSPSMLS--
EVPPSLSHILFLFGLLNAPLDPLLYGAFTLGCRR-------------
>gnrhr3_Tetraodon_nigroviridis [EMBL: Q3LG36]
--DALQLPTFSTAAKVRVIITFSLCAVSAVCNLVVLWAAGNG---G----
KRKSHVRILIMNLTVADLLVTFIVMPVDAVWNIT---VQWQAGD-----------------------------------
AACRLLMFLKLVAMYSCAFVTVVISLDRQSAILNPLGISEAK--
RKSKITLAVAWTMSVVLSLPQMFIFHNVTITVPE-----------------NFTQCTTHG---SFVQHWQETLYN--
MFTFACLFLLPLIIMIFCYTRILVEISSRMAR-----NNLLSRDVHLRRSHNN-----------------------------
IPKARMRTLKMSIVIVTSFIVCWTPYYLLGLWYWLFPEKME-
ETVSHSLTHMLFIFGLFNACLDPITYGLFTVRLHQGLKR---------
>gnrhr2_Gallus_gallus [EMBL: Q5EFE0]
AEEPLLLPTFSPAAQARVAATFVLFVLSAGCNVAVLRAAGGRR--G----
GGRSHIRVLLRHLAAADLLVTVVVMPLDAIWNIT---LQWRAGD-----------------------------------
LACRLLMYLRLLAMYASAFVTVVISLDRQAAILRPLAIARAR--
CRNRAMLRAAWMLSAALAVPQLFLFHTVTLHAPH-----------------NFTQCTTHG---SFPQPWHETLYN--
MLSFSCLFLLPLLIMVCCYTRILLEISRRMGS-----SLFSSRDVPLRCSGSN-----------------------------
IPRARLRTLKMSLVIVSSFILCWTPYYLLGLWYWFCPRAMQ-
QKVPPSLSHILFIFGLFNACLDPITYGLFTIPFRRRCGCP--------
>vasr2_Mus_musculus [EMBL: Q3SWS4]
------------LVRAELALLSTIFVAVALSNGLVLGALIRRGR-----
RGRWAPMHVFISHLCLADLAVALFQVLPQLAWDAT---DRFHGPD-----------------------------------
ALCRAVKYLQMVGMYASSYMILAMTLDRHRAICRPMLAYRHGGGARWNRPVLVAWAFSLLLSLPQLFIFAQRDVGNGS-----

------------GVFDCWARF-----AEPWGLRAYV--
TWIALMVFVAPALGIAACQVLIFREIHASLVPGPSERAGRRRR---GHRTGSPSEG------------------------
AHVSAAMAKTVRMTLDPPLCCSCCWLALTAVPTPGSMLPS----------------
VAVSPRSCVACFAVLRGTPHTAWVLKMSP-------
